# Ability of real-time PCR for differential diagnosis of various forms of cutaneous leishmaniasis: a comparative study with histopathology

**DOI:** 10.1186/s13104-019-4666-5

**Published:** 2019-09-23

**Authors:** Maryam Fekri-SoofiAbadi, Meisam Fekri, Alireza moradabadi, Reza Vahidi, Simin Shamsi-Meymandi, Donya Dabiri, Shahriar Dabiri

**Affiliations:** 1 Pathology and Stem Cell Research Center, Department of Pathology, Afzalipour Medical School, Kerman University of Medical Sciences, 22 Bahman Blvd, 7616913555 Kerman, Iran; 2 Dermatology Department, Afzalipour Hospital, Kerman University of Medical Sciences, Kerman, Iran; 3 Department of Medicine, Montefiore New Rochelle Hospital, Albert Einstein College of Medicine, New York, USA

**Keywords:** Leishmaniasis, Real-time PCR, Ridley scoring system

## Abstract

**Objective:**

Histopathological studies suggest that parasite load is different between acute and chronic forms of cutaneous leishmaniasis (CL). However, highly sensitive detection methods are still needed to distinguish different forms of leishmaniasis. In the present study, we developed a quantitative real-time polymerase chain reaction (PCR) to detect and quantify *Leishmania tropica* parasites in paraffin-embedded tissue samples.

**Results:**

The ability of real-time PCR for leishmania detection was higher than histopathological evaluation. The quantitative real-time PCR (qPCR) quantified parasite loads were highly correlated with microscopic results (*r *= 0.598; *P *< 0.001). Among patients, the parasite load was inversely correlated with disease duration (acute CL lesions had very higher parasite load than chronic CL lesions), but there was no difference in the parasite load according to the patients’ age and sex as well as location of the lesions. In contrast to Ridley scoring system (P < 0.001), there were no statistically significant differences in the relative number of parasites among the lupoid and non-lupoid forms of chronic lesions in real-time PCR (P = 0.549), which indicates the superiority of histopathological evaluation for chronic forms differentiation.

## Introduction

Dry cutaneous leishmaniasis (CL) caused by *Leishmania tropica* is a significant parasitic disease in Iran [1]. The clinical phenotype, histopathology, and the number of organisms are diverse among acute, chronic lupoid, and chronic non-lupoid forms of this infectious disease [2]. In histopathology of acute CL, plasma cells, histiocytes, epithelioid cells, and occasionally eosinophils and giant cells, and dense dermal infiltrate of lymphocytes are seen. Also, numerous intracytoplasmic Leishman bodies parasitized macrophages and sometimes neutrophils are seen throughout the reticular dermis. A small number of infected macrophages and multifocal small tuberculoid granulomas composed of epithelioid cells, histiocytes, and occasional giant cells are seen more in chronic form. In addition, mild to moderate mononuclear infiltrates (lymphocytes and plasma cells) adjacent to the granuloma along with fibrosis and telangiectasia are present. Low numbers of organisms, erythematous papules at the periphery of a scar of a healed acute lesion, and granulomas consisting of tubercles surrounded by lymphocytes, histiocytes, and giant cells are the most pathological findings in the lupoid forms of the disease; although, because of scant organisms in cutaneous lesions specifically in chronic leishmaniasis, microscopic studies has less sensitivity [2–9].

Laboratory diagnosis of CL relies on either the microscopic detection of Leishman bodies in cutaneous tissue or the culture and isolation of parasites from lesions biopsy samples [10, 11]. Apart from high specificity, inadequate sensitivity, difficulty, and time consuming nature are among disadvantages of these methods [12]. Nowadays, PCR-based testing of skin lesion biopsies is known as a sensitive and specific test for diagnosis and quantification of leishmaniasis [13–16]. The analysis of the load of leishmania parasites within the skin lesions would be important not only for diagnostic purposes, but also for an eventual follow-up of a patient’s response to treatment [17]. Accordingly, in the present study we applied a standardized qPCR assay to detect *Leishmania tropica* load in paraffin blocks of various CL forms. The differentiation ability of this quantitative method was compared with semi-quantitative pathological scoring system.

## Main text

### Materials and methods

#### Patients and sampling

Forty patients presenting with acute (n = 10), chronic lupoid (n = 16), and chronic non-lupoid (n = 14) forms of CL who were referred to the Dermatopathology Department of Afzalipour Hospital (2010–2013) were selected to participate in our study. Patient selection was performed after evaluation of inclusion/exclusion criteria. The patients were considered to be included in the study if they have confirmed and long-term CL (≥ 3 years), had received at least 3 times glucantime treatment, and were able to give contact information for the follow-up. We excluded patients with other skin diseases or with small biopsy samples.

#### Histopathology

Skin biopsies were fixed in formalin, routinely processed, and after embedding in paraffin, sections were stained with hematoxylin and eosin, based on general approach. After grouping of cutaneous lesions according to Azadeh [18] classification (Anergic macrophage reaction, Focalized histiocytic reaction, Diffuse necrotizing reaction, Diffuse lympho-histiocytic reaction, and Lupoid granulomatous reaction), the Ridley scoring system [19] was applied for determination of parasite load, as follows from 0 to + 4:

- 0: None amastigote
- + 1: One or more amastigotes
- + 2: 10 or more amastigotes
- + 3: 100 or more amastigotes
- + 4: 1000 or more amastigotes.

In should be noted that for uniform inflammatory cell-counting in all samples, it was decided to count the cells in inflammatory centers near the parasite and around granulomas. In addition, histopathologic alterations including necrosis, unorganized or organized granuloma, cellular (polymorphonuclears, eosinophils, giant cells, lymphocytes, plasma cells, and macrophages) infiltration and parasite index were estimated through an arbitrary semiquantitative procedure.

#### DNA extraction

For DNA extraction, 5 μm sections from paraffin-embedded blocks were cut using disposable blades and deparaffinized by hot xylene and then, were hydrated (descending grades of alcohol) and incubated in proteinase K (20 μg/μL, at 60 °C). After digestion completed (3 days), the DNA was isolated using a QIAamp^®^ DNA Mini Kit (QIAGEN, 51304), according to the manufacturer′s protocol.

#### Real-time PCR assay

We applied a probe-based assay targeting rRNAITS region to detect and quantify parasites in the samples (Table 1). PCR amplification reaction was fulfilled using ABI StepOne system (Applied Biosystems, USA) and in a 25 μL of reaction mixture, containing 12.5 μL of master mix, 2 μL of forward and reverse primers for beta-actin and rRNAITS regions, 1.5 μL probe, 2 μL of H_2_O, and 5 μL of extracted DNA. Thermal cycling conditions started at 95 ^°^C for 2 min followed by 95 ^°^C for 20 s (denaturation), and 60 ^°^C for 30 s (annealing and extension), which were programmed for 45 cycles. A cycle threshold (Ct) for each sample was determined based on the required cycles for the fluorescent signal to cross the background level.

**Table 1:** Primers used in our study

Primers	Sequences (5′–3′)
L.ITS.F	5′-CAAATACACGCATGCACTCTC-3′
L.ITS.R	5′-TTTAATAATCCTGGTCACAGCC-3′
L.ITS.P	FAM-5′AGCGTCGAAACTCCTCTCTGGTGC3′-TAMRA
Actin.F	5′-ACCACCTTCAACTCCATCATG-3′
Actin.R	5′-CTCCTTCTGCATCCTGTCG-3′
Actin.P	JOE-5′ ACATCCGCAAAGACCTGTACGCC 3′-TAMRA

*F* forward, *R* reverse, *P* probe, *L.ITS* leishmania ITS (internal transcribed spacer) gene

#### Quantification of parasite DNA load

For absolute quantification, the standard strain (MHOM/Sudan/58/OD) of *L. tropica* was cultured in RPMI1640 medium and serial dilutions (10 to 10^7^) were prepared. Subsequently, a standard curve was set by plotting the Ct values against different standards with known concentration of the parasite’s DNA.

#### Statistical analysis

The differences between experimental groups were analyzed using the ANOVA (Tukey test). The Spearman’s rank correlation coefficient was used for evaluation of the relationship between real-time PCR and histopathological results. The SPSS software (version 22) was applied in this study.

### Results

Histopathology and real-time PCR results in studied patients with different forms of CL are summarized in Table 2 and 3. Forty patients with confirmed CL were enrolled: 25 (62.5%) men and 15 (37.5%) women, with mean age of 32 years (range 6–73 years). To evaluate the correlation between the qPCR assay and *histopathological evaluation*, collected samples were analyzed in parallel by both methods. The linearity of qPCR results was approved (diagram slope of − 3.23 and correlation coefficient (r^2^) ≥ 0.997) [20]. This assay allowed the quantification of the parasite load in all samples, while the microscopic evaluation allowed this in 32 samples (80%, 8 negative samples corresponded to lupoid patients), which is indicating that the former method is more sensitive than the latter.

**Table 2:** Summary of patients informations

Cutaneous leishmaniasis forms	Number of patients	Ridley score					Real-time PCR		P value (Ridley)			P value (qPCR)		
		0^+^	1^+^	2^+^	3^+^	4^+^	C_t_ (mean ± SD)	Parasite load (mean)	A	CL	CNL	A	CL	CNL
A	10	–	–	4	4	2	26.24 ± 4.59	13,064	–	< 0.001	0.038	–	< 0.001	< 0.001
CL	16	10	6	–	–	–	31.88 ± 0.24	63	< 0.001	–	< 0.001	< 0.001	–	0.549
CNL	14	–	4	7	3	–	31.31 ± 0.71	98	0.038	< 0.001	–	< 0.001	0.549	–

*A* acute form, *CL* chronic lupoid form, *CNL* chronic non-lupoid form

**Table 3:** Detailed analysis of each patient

Patient	Form	Lesion site	Ridley score	Parasite load with qPCR
1	A	Hand	2^+^	63,096
2	A	Hand	3^+^	16,982
3	A	Lower lip	3^+^	4163
4	A	Hand	4^+^	5495
5	A	Hand	3^+^	3708
6	A	Face	2^+^	1942
7	A	Face	2^+^	6598
8	A	Hand	2^+^	2459
9	A	Lower lip	3^+^	1485
10	A	Face	4^+^	24,712
11	CNL	Forearm	3^+^	186
12	CNL	Hand	1^+^	89
13	CNL	Ankle	1^+^	161
14	CNL	Nose	2^+^	78
15	CNL	Hand	1^+^	72
16	CNL	Hand	2^+^	56
17	CNL	Face	3^+^	115
18	CNL	Leg	1^+^	76
19	CNL	Ankle	2^+^	85
20	CNL	Forearm	2^+^	74
21	CNL	Hand	2^+^	90
22	CNL	Face	2^+^	96
23	CNL	Hand	2^+^	84
24	CNL	Forearm	3^+^	110
25	CL	Hand	1^+^	48
26	CL	Hand	0	59
27	CL	Forearm	1^+^	39
28	CL	Face	0	95
29	CL	Face	0	68
30	CL	Hand	0	91
31	CL	Hand	0	64
32	CL	Hand	1^+^	59
33	CL	Face	0	72
34	CL	Face	1^+^	87
35	CL	Hand	1^+^	99
36	CL	Forearm	1^+^	68
37	CL	Face	0	47
38	CL	Face	0	40
39	CL	Face	0	37
40	CL	Hand	0	35

*A* acute form, *CL* chronic lupoid form, *CNL* chronic non-lupoid form, *M* male, *F* female

As presented in Tables 2 and 3, acute form has higher parasite load than chronic ones (*P *< 0.001) by real-time PCR. The mean parasite load in chronic lesions (*n *= 30) was 0.08 × 10^3^ parasites, compared with 13.064 × 10^3^ in acute lesions (*n *= 10, *P *< 0.001). Interestingly, there was no significant difference in parasite load among lupoid and non-lupoid lesions by real-time PCR (*P *= 0.549). According to histopathological analysis, there were statistically significant differences in the relative number of parasites among the acute and chronic (P< 0.01) and chronic-lupoid and non-lupoid forms (*P *< 0.001). These results indicate the superiority of histopathological evaluation (Ridley scoring system) for differentiation of various forms of CL.

### Discussion

In order to accurately and confidently quantify parasites in paraffin-embedded biopsy samples, we evaluated the parasitic load in acute and chronic forms using real-time PCR and histopathological scoring system. The focus of the present study was to compare the diagnostic ability of two common methods in a relatively large number of patients with CL. The power of the used qPCR assay [21] has allowed the quantification of a broad range of parasite load levels in tissue lesions. In terms of diagnostic sensitivity, our results confirmed that the sensitivity of real-time PCR is indeed higher than histopathological scoring system. Our findings are also consistent with the findings of previous studies that focused on different abundance of parasite in various forms of CL, pointing to inversely correlation of parasite load with the disease duration. Namely, in both methods of this study, acute form has higher parasite load than chronic ones. Interestingly, in contrast to Ridley scoring system (P < 0.001), there were no statistically significant differences in the relative number of parasites among the lupoid and non-lupoid forms of chronic lesions in real-time PCR (P = 0.549), which indicates the superiority of histopathological evaluation in differentiation of chronic forms. It should be noted that the analysis performed here revealed no significant differences in parasite load with regard to the age, sex, and location of skin lesions. These findings were consistent with other studies [22–25]. For example, Mashayekhi et al. in a study on 11 male and 9 female patients with a mean age of 17.5 years showed that PCR was positive in 60% of the samples and no correlation was found between the results of PCR and age, sex, duration, and location of the lesions [26]. Venkataram et al. indicated that 65% of acute, subacute, and chronic lesions manifested leishmania parasites in tissues. But they could not find the relationship between the duration of lesions and PCR results [25]. Weigle and others showed that PCR sensitivity was higher than the conventional assays for the diagnosis of acute lesions while for chronic samples, the sensitivity of PCR was much higher than the conventional assays [27]. In this regard, Verma et al. conducted real-time assay to estimate parasite burden in clinical samples of visceral leishmaniasis and patients with post kala-azar dermal leishmaniasis. Concurrent diagnostic and prognostic ability of this assay, provide a simple molecular instrument to detect parasite and show the efficacy of anti-leishmanial drugs or vaccines [28]. In line with this, Dabiri et al. compared the effect of different treatments on DNA load of leishmania using real-time PCR method [29]. Jara et al. improved a quantitative real-time PCR (qPCR) method targeting mini-circle kinetoplast DNA (kDNA) to find and quantify *Leishmania* (*Viannia*) parasites. According to the parasite species, the patients’ age, and number or area of lesions, there was no difference in parasite load [17]. Sirian et al. conducted a comparison between conventional, molecular, and immunohistochemical methods for CL detection and reported that immunohistochemical and molecular techniques were more sensitive [30–32].

Our observations support the validity of using real-time PCR to simultaneously detect and quantify the leishmania load in human lesions, particularly in chronic lesions. This highly sensitive quantitative technique [10, 20, 21] can be employed also for monitoring the parasite load during treatment and follow-up as a way to assess the outcome of treatment.

## Limitations

Accurately and confidently quantify parasites in biopsy samples help to evaluated the parasitic load in acute and chronic forms using real-time PCR in scoring CL. Using the paraffin-embedded biopsy samples make our samples collection time short. But it’s make the DNA extraction laboratories and effect on the quality of extracted DNA.

## Data Availability

Please contact corresponding author (S.D) for data requests.

## Abbreviations

CL: cutaneous leishmaniasis
PCR: polymerase chain reaction
Ct: cycle threshold
CNL: chronic non-lupoid form
qPCR: quantitative real-time PCR
